# Detection of Copy Number Variants Reveals Association of Cilia Genes with Neural Tube Defects

**DOI:** 10.1371/journal.pone.0054492

**Published:** 2013-01-17

**Authors:** Xiaoli Chen, Yiping Shen, Yonghui Gao, Huizhi Zhao, Xiaoming Sheng, Jizhen Zou, Va Lip, Hua Xie, Jin Guo, Hong Shao, Yihua Bao, Jianliang Shen, Bo Niu, James F. Gusella, Bai-Lin Wu, Ting Zhang

**Affiliations:** 1 Capital Institute of Pediatrics, Beijing, China; 2 Department of Laboratory Medicine, Children's Hospital Boston, Boston, Massachusetts, United States of America; 3 Center for Human Genetic Research, Massachusetts General Hospital, Boston, Massachusetts, United States of America; 4 Shanghai Children's Medical Center, Jiaotong University, Shanghai, China; 5 Harvard Medical School, Boston, Massachusetts, United States of America; 6 Institute of Acu-moxibustion, China Academy of Chinese Medical Sciences, Beijing, China; 7 Department of Pathology, Capital Institute of Pediatrics, Beijing, China; 8 Department of Hematology, Navy General Hospital of PLA, Beijing, China; 9 Department of Biotechnology, Capital Institute of Pediatrics, Beijing, China; 10 Children's Hospital and Institutes of Biomedical Science, Shanghai Medical College, Fudan University, Shanghai, China; The Chinese University of Hong Kong, Hong Kong

## Abstract

**Background:**

Neural tube defects (NTDs) are one of the most common birth defects caused by a combination of genetic and environmental factors. Currently, little is known about the genetic basis of NTDs although up to 70% of human NTDs were reported to be attributed to genetic factors. Here we performed genome-wide copy number variants (CNVs) detection in a cohort of Chinese NTD patients in order to exam the potential role of CNVs in the pathogenesis of NTDs.

**Methods:**

The genomic DNA from eighty-five NTD cases and seventy-five matched normal controls were subjected for whole genome CNVs analysis. Non-DGV (the Database of Genomic Variants) CNVs from each group were further analyzed for their associations with NTDs. Gene content in non-DGV CNVs as well as participating pathways were examined.

**Results:**

Fifty-five and twenty-six non-DGV CNVs were detected in cases and controls respectively. Among them, forty and nineteen CNVs involve genes (genic CNV). Significantly more non-DGV CNVs and non-DGV genic CNVs were detected in NTD patients than in control (41.2% *vs.* 25.3%, *p*<0.05 and 37.6% *vs.* 20%, *p*<0.05). Non-DGV genic CNVs are associated with a 2.65-fold increased risk for NTDs (95% CI: 1.24–5.87). Interestingly, there are 41 cilia genes involved in non-DGV CNVs from NTD patients which is significantly enriched in cases compared with that in controls (24.7% *vs.* 9.3%, *p*<0.05), corresponding with a 3.19-fold increased risk for NTDs (95% CI: 1.27–8.01). Pathway analyses further suggested that two ciliogenesis pathways, tight junction and protein kinase A signaling, are top canonical pathways implicated in NTD-specific CNVs, and these two novel pathways interact with known NTD pathways.

**Conclusions:**

Evidence from the genome-wide CNV study suggests that genic CNVs, particularly ciliogenic CNVs are associated with NTDs and two ciliogenesis pathways, tight junction and protein kinase A signaling, are potential pathways involved in NTD pathogenesis.

## Background

Neural tube defects (NTDs) are common and severe birth defects. They arise between the third and fourth week of embryogenesis because of partial or complete failure of neural tube closure [1]. Severe NTDs, such as craniorachischisis and anencephaly, are directly related to high morbidity and mortality. Less severe forms, such as open spina bifida, lead to life-long disabilities and impose a tremendous burden on affected families [1]. Approximately 1 in 1,000 pregnancies worldwide is affected by NTD [2]. The prevalence of NTDs is high in China, with approximately 27.4 per 10,000 pregnancies reported [3]. In particular, the Shanxi Province in China has the highest NTD occurrence at the rate of 138.7–199.4/10,000 [4], [5].

NTDs are considered to represent a complex disorder as both environmental and genetic factors contribute to NTDs [6]. It has been estimated that up to 70% of human NTDs can be attributed to genetic factors [7]. The recurrence risk of NTDs for siblings of indexed cases is 2–5%, which is approximately 50-fold higher than that of general population [8]. The robust NTD phenomena in gene knockout mouse models imply the existence of high penetrance NTD genes [9], [10]. A number of candidate genes from mouse NTDs were screened in humans, but so far only the *MTHFR* polymorphism and pathogenic mutations of the planar cell polarity (PCP) core gene were shown to be consistently associated with human NTDs [11], [12], [13], [14], [15], [16], [17], [18], [19]. These PCP gene mutations only account for 0.75–22.2% of different isolated NTDs, with low mutation rate in spinal and a higher rate in cranial NTDs [19], [20]. On the other hand, conventional karyotyping has revealed microscopic chromosomal abnormalities in 2.5–10.26% of fetal and newborn with NTDs [21], [22], [23], [24], suggesting that genomic imbalances are one of the genetic bases of NTDs. No specific NTD candidate gene has been implicated by cytogenetic studies due to low resolution of conventional karyotyping. With the advent of chromosomal microarray technology, we anticipate detection of smaller NTD-specific copy number variants (CNVs) that could reveal novel candidate genes and pathways associated with NTDs.

## Methods

### Study Design

A hospital-based case-control study was conducted from April 2006 to December 2008 in the Lvliang region of Shanxi Province. All pregnant women who enrolled this study provided their written informed consent to participate in this study. The study protocol was reviewed and approved by the Ethics Board of Capital Institute of Pediatrics.

### Samples Recruitment

Collection of NTD-affected embryos took place during routine prenatal checkups in multiple local county hospitals. Detailed records of gestational ages, general development of the embryo, and embryo B-ultrasound data were kept by obstetricians. The epidemiological method was described in detail in our previous publication [25]. Medical abortions were carried out by obstetricians to terminate NTD-affected pregnancies. Pathologic diagnosis of NTDs was performed by an experienced pathologist according to the International Classification of Disease, Tenth Revision, codes Q00.0, Q05.9 and Q01.9 (http://apps.who.int/classifications). The embryos aborted for non-medical reasons from same region were also enrolled and used as matched controls. Routine prenatal checkups, questionnaire interviews and autopsies were completed for controls, and any embryos that harbored any pathological malformation or intrauterine growth retardation were excluded from control group.

### Array-comparative Genomic Hybridization (Array-CGH)

Brain tissue (25 mg) from the cerebrum was used for DNA extraction (DNeasy Blood & Tissue kit, Qiagen, Valencia, CA). For the cases with anencephaly, residue brain tissue in the cavity of the skull or spinal tissue was collected for DNA extraction. Array-CGH was performed according to previously published methods [26] using the Agilent 244 K Oligonucleotide CGH microarray platform (Agilent Technologies Inc., Palo Alto, CA) in the Genetic Diagnostic Laboratory at Children’s Hospital Boston.

### Workflow for the Identification of CNVs

Deletions and duplications were identified using at least five consecutive probes by the DNA Genomic Workbench Standard Edition 5.0.14 software (Agilent Technologies Inc., Palo Alto, CA). We applied a minimal size cutoff (>30 Kb) for CNV detection. A CNV was defined as non-DGV when it does not overlap, or overlaps less than 20% with reported CNVs in the Database of Genomic Variants (DGV, http://projects.tcag.ca/variation/). These CNVs were also compared with the CNVs detected in normal Chinese populations [27], [28]. CNVs that involve one or more Refseq genes or exonic region of a gene were defined as genic CNVs [29]. CNVs containing cilia genes were termed as ciliogenic CNVs.

### Validation of Selected CNVs

Twenty randomly selected CNVs were validated by long-range PCR (for heterozygous deletions) and real-time quantitative PCR (for deletions and duplications). Multiple breakpoint-specific primer pairs were used for long-range PCR (Platinum PCR SuperMix High Fidelity kit, Invitrogen Corp. 12532-016) until 1–2 kb unique amplicons were generated and subsequently sequenced.

Three primer pairs targeting the center and two sides of a CNV were designed for real-time quantitative PCR (Applied Biosystems, Carlsbad, CA). A normal male was used as a reference control and the *ß-actin* gene was used as an internal control. The assays were done in triplicate for each CNV.

### Ingenuity Pathway Analysis

We uploaded the gene list generated from all non-DGV genic CNVs to the online Ingenuity Pathway Analysis platform (IPA, Ingenuity Systems Analysis, www.ingenuity.com) for identifying associated networks, related disorders and canonical pathways. Each identifier was mapped to its corresponding object in Ingenuity’s Knowledge Base. Fischer’s exact test was used to calculate a *p-*value that determined the probability of the genes involved in each pathway. A gene list containing 223 NTD candidate genes (Table S5) after literature review [9], [10], [30], [31], [32] was also analyzed using the same setting.

### *MKS1, MKS3(TMEM67)* Mutation Screening

Meckel-Gruber Syndrome (MIM 249000) is a major cause of systemic NTD [24]. To rule out the involvement of Meckel-Gruber Syndrome, we did the mutation screening for *MKS1* (MIM 609883) and *MKS3/TMEM67* (MIM 609884) in 38 systemic NTD cases presenting encephalocele and/or urinary development malformation [33]. The coding exons and intronic flanking regions of *MKS1/MKS3* were amplified and sequenced. Sequences were analyzed using Mutation Surveyor V3.30 (SoftGenetics, State College, PA). The sequences of primers designed for 42 amplicons were listed in the Table S6.

### Statistical Analysis

Data were analyzed using SPSS version 10.0 statistics software (SPSS Inc., Chicago, IL). Normal distribution of CNV sizes was verified by the Kolmogorov-Smimov method. Fisher’s exact test or the **χ** ^2^ test was used for categorical variables, Student’s *t* test was used for continuous variables, and Wilcoxon rank-sum test was used for rank categorical variables. Logistical regression was used to determine adjusted odds ratios and their associated 95% confidence intervals. A value of *p<*0.05 (two-tailed) was considered significant.

## Results

### Non-DGV CNVs were Enriched in NTD Samples

A total of 85 cases (NTD-affected embryos) and 75 controls were collected. No case had the positive familial NTD history, suggesting they all were sporadic NTDs. The detailed categories of NTD cases were listed on Table 1. No known pathogenic or novel, rare mutation of *MKS1/MKS3* genes was detected.

**Table 1:** The general characteristics of the NTD cohort.

	N	Cranial NTDs (%)	Spinal NTDs (%)
**Ratio of male/female**	45/40*		
** Isolated NTDs**	22	12 (54.5%)	10 (45.5%)
** Systemic NTDs**	63	39 (61.9%)	24 (38.1%)
**Total**	85	51	34

* p = 0.650, compared to controls including 39 male and 36 female.

**Figure 1:**
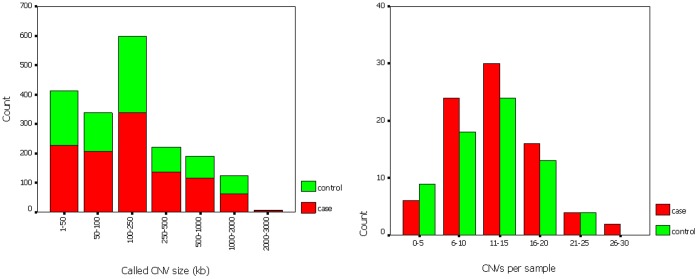
Summary of whole genomic copy number variants (CNVs) in the two groups; Wilcoxon rank-sum test was used for statistical analysis. Figure 1A: Distribution of all called genomic CNVs by size. Figure 1B: Distribution of all called genomic CNVs by frequency.

A total of 1057 CNVs in 85 cases and a total of 821 CNVs in 75 controls were detected. The per sample CNV load either by count or by size was not different between cases and controls (CNV count per sample: 12.89 in cases *vs.* 12.07 in controls, *p*>0.05; CNV size per sample: 283 kb in cases *vs.* 330 kb in controls, *p*>0.05). Stratifying CNVs by size or grouping samples by CNV count did not show any significant difference among cases and controls either (Figure 1A and 1B).

After filtering out benign CNVs reported in DGV and Chinese-specific common CNVs (four in cases and five in controls) [27], [28], 81 non-DGV CNVs were identified, 55 in NTD cases and 26 in controls. We compared this non-DGV CNV list with the clinical diagnostic CNV dataset in Genetic Diagnostic laboratory, Boston Children’s Hospital and a Chinese CNV dataset consisting of about 300 mental retardation patients and their unaffected relatives. None of the non-DGV CNVs identified from NTD cases was present in these two datasets, suggesting that these non-DGV CNVs are very rare and/or NTD-specific. Out of 81 non-DGV CNVs, 59 CNVs (40 in NTD cases and 19 in controls) involve one or more genes (termed “genic CNVs”). Six different-sized genic CNVs validated by PCR are shown in Figure S1 (Table S1 for detailed primer information).

The average count of non-DGV CNVs per sample was 1.85-fold higher in NTDs than in controls (0.65 *vs.* 0.35) while the average count of non-DGV genic CNVs per sample was 1.88-fold higher in case than in controls (0.47 *vs.* 0.25). The proportions of samples with non-DGV CNVs and genic CNVs were significantly higher in cases than in controls (41.2% *vs.* 25.3%, *p*<0.05; 37.6% *vs.* 20%, *p*<0.05, see Table 2). Non-DGV genic CNVs had a lower *p* value than non-DGV CNVs (0.01 *vs.* 0.03, labeled *** in Table 2). Logistical regression analysis showed that non-DGV CNVs and genic CNVs lead to an increased risk for NTDs (non-DGV CNVs, OR = 2.26, CI = 1.10–4.74; non-DGV genic CNVs, OR = 2.65, CI = 1.24–5.87). Furthermore, for samples without non-DGV CNVs, we compared the whole genomic common CNV burden in cases and controls. Neither the count nor the size of genome-wide common CNVs was different in cases and controls (CNV count: 11.61 *vs.* 11.92, *p*>0.05; CNV size: 292 kb *vs.* 344 kb, *p*>0.05). The above analysis indicated that it is the non-DGV genic CNVs, not common CNVs that are associated with increased risk of NTDs. We also compared the non-DGV CNV size in NTDs and controls and no difference was identified (Table S2).

**Table 2:** Comparison of non-DGV CNVs, non-DGV genic CNVs and ciliogenic CNVs in NTD cases and controls.

	NTDs N (%)	Control N(% )	*p* value (2-tail)	OR**
**Number of samples**	85	75		
**Count of non-DGV CNVs**	55	26		
**Count of non-DGV genic CNVs**	40	19		
**Count of gene compassed in non-DGV CNVs**	138	44		
**Count of cilia gene compassed in non-DGV genic CNVs**	41	12		
**Samples with non-DGV CNVs** *	35/85 (41.2%)	19/75 (25.3%)	0.034	2.26 (1.10–4.74)
** Samples with non-DGV deletion**	23/85 (27.1%)	11/75 (14.7%)	0.056	
** Samples with non-DGV duplication**	21/85 (24.7%)	12/75 (16.0%)	0.170	
**Non-DGV CNV count per sample**	0.65 (55/85)	0.35 (26/75)		
**Samples with non-DGV genic CNVs** *	32/85 (37.6%)	15/75 (20.0%)	0.014***	2.65 (1.24–5.87)
** Samples with non-DGV genic deletion**	19/85 (22.4%)	9/75 (12.0%)	0.085	
** Samples with non-DGV genic duplication**	18/85 (21.2%)	10/75 (13.3%)	0.190	
**Non-DGV genic CNV count per sample**	0.47 (40/85)	0.25 (19/75)		
**Samples with non-DGV ciliogenic CNVs** *	21/85 (24.7%)	7/75 (9.3%)	0.011***	3.19 (1.27–8.01)
** Samples with non-DGV ciliogenic deletion**	10/85 (11.8%)	2/75 (2.7%)	0.036	
** Samples with non-DGV ciliogenic duplication**	12/85 (14.1%)	5/75 (6.7%)	0.130	
**Non-DGV ciliogenic CNV count per sample**	0.29 (25/85)	0.09 (7/75)		

* For sample with both deletion and duplication, we counted twice times, It was same for genic CNVs and ciliogenic CNVs;

** Logistical regression analysis among different CNVs and NTDs;

*** Lower *p* value in non-DGV genic CNVs and ciliogenic CNVs compared with non-DGV CNVs;

### Enrichment of Ciliogenic CNVs in NTDs

The total number of genes involved in NTD-specific CNVs was about 3-fold that in controls (138 *vs.* 44, see Table S3 and S4). Importantly, nine genes (*CALR3*, *CTNNA3*, *CYP27B1*, *LMO1*, *RAB19, RABL5*, *SLC* gene family, *ZIC*, *WNT*) in NTD-specific CNVs are homologous to genes that have been reported as NTD candidate genes in mouse models (labeled yellow in Table S5), suggesting that a subset of genes in NTD-specific CNVs is directly contributing to the pathogenesis of NTDs in some samples. After referring to recently published literature reporting 828 ‘ciliome’ genes [34], forty one out of 138 genes from NTD-specific CNVs were identified as cilia genes. In controls, only twelve cilia genes were influenced by non-DGV CNVs.

**Figure 2:**
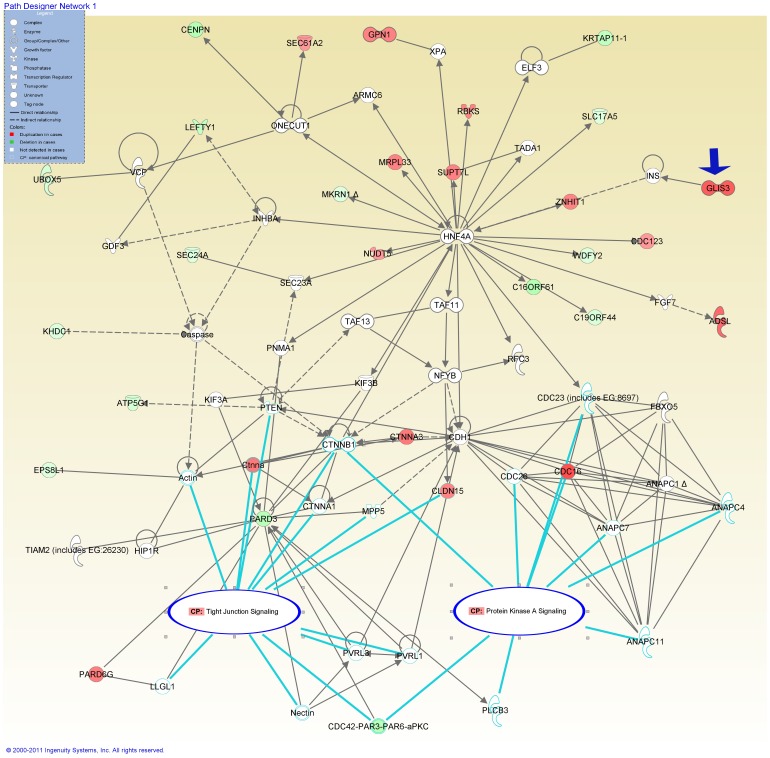
Top bio-functional networks in NTD-affected cases. The network “*Cell Morphology, Developmental Disorder, Skeletal and Muscular Disorders*” was identified in genes from NTD-specific CNVs by IPA (red indicates the duplication CNV, and green indicates deletion CNV). Genes are represented as nodes. Edges indicate known interactions between proteins (solid lines for direct interactions, dashed lines for mean indirect interactions). The gene shapes are indicative of molecular class. The canonical pathways (CP in the oval regions) were Tight junction signaling and Protein kinase A signaling.

IPA core analysis showed that bio-functional networks, related diseases/disorders, and canonical pathways were all different between gene lists from cases and controls (Table 3). In cases, the top-ranked network was related to “*Cell Morphology, Developmental Disorder, Skeletal and Muscular Disorders*”. Twenty-eight genes from cases (see Figure 2, green indicates deletion, red indicates duplication) involved in this network. The top related diseases and disorders involved were *“Genetic and Neurological Disease”*. The canonical pathway analysis revealed that “tight junction signaling” and “protein kinase A signaling” were two significant pathways involved in genes from NTD-specific CNVs (Figure 2, labeled as CP in the oval regions). The *PAR3-PAR6-aPKC* complex and *CTNNB1* were shared components of the two canonical pathways.

**Table 3:** Top networks, related disorders and diseases identified in NTD case, controls and known NTD candidate genes.

Bio-function networks		Score	Genes
NTDs	Cell Morphology, Developmental Disorder, Skeletal and Muscular Disorders	51	28 in Figure 2
	Renal and Urological Disease, Metabolic Disease, Genetic Disorder	38	
	Cell Cycle, Cellular Development, Cellular Growth and Proliferation	35	
Controls	Cellular Function and Maintenance, Cell Death, Gene Expression	49	
	Cell Death, Lipid Metabolism, Small Molecule Biochemistry	14	
Known NTD candidate genes	Embryonic Development, Neural System Development and Function, Organ Development	47	33 in Figure 3
	Amino Acid Metabolism, Molecular Transport, Small Molecular Biochemistry	37	
	Developmental Disorder, Neurological Disease, Skeletal and Muscular Disorders	36	
	Molecular Transport, Drug Metabolism, Lipid Metabolism	32	
	Carbohydrate Metabolism, Tissue Development, Embryonic Development	31	
**Disease and disorders**		**P values**	**Genes**
NTDs	Genetic Disorder	4.72E−04–4.58E−02	56
	Neurological Disease	4.72E−04–4.77E−02	35
	Endocrine System Disorders	1.06E−03–2.61E−02	24
	Metabolic Disease	1.06E−03–4.58E−02	27
Controls	Immunological Disease	8.87E−04–2.05E−02	5
	Infectious Disease	8.87E−04–8.87E−04	4
Known NTD candidate genes	Developmental Disorder	1.10E−122–9.33E−07	154
	Neurological Disease	1.10E−122–4.70E−09	113
	Skeletal Disorder	2.68E−98–6.49E−07	101
	Genetic Disorder	8.48E−39–1.08E−06	76

We further identified that 12 cilia genes (*GLIS3, CENPN, CDC123, WDFY2, C16orf61, SLC17A5, CTNNA3, CLDN15, CDC16, ATP5G1, PARD3* and *PARD6G*) from 10 cases participate this top network (see Figure 2 for their locations in the network). Both the core gene of the network-*HNF4A* and shared components (*PARD3, PAR6D6G* and *CTNNB1*, see Figure 2) belong to cilia genes. In order to further evaluate the involvement of cilia genes in NTDs, we compared the frequency of non-DGV CNVs containing cilia genes (termed as non-DGV ciliogenic CNVs) in NTD cases and controls. In NTDs, 21 cases carry non-DGV ciliogenic CNVs, which was significant higher than that in controls. (24.7% *vs.* 9.3%, *p*<0.05, see Table 2). Non-DGV ciliogenic CNVs led to a 3.19-fold (95% CI: 1.27–8.01) increased risk for NTDs. In order to further explore the association of non-DGV ciliogenic CNVs with subtypes of NTD (Table 4), NTD cases were stratified into cranial or spinal NTDs, isolated or systemic NTDs according to patient phenotypes. Table 4 shows that the frequency of ciliogenic CNVs was not significantly higher in cranial NTDs than in spinal NTDs (21.6% *vs*. 29.4%, *p*>0.05). Meanwhile, the frequency of ciliogenic CNVs was not significantly higher in systemic NTDs compared with isolated NTDs (28.6% *vs.* 13.6%, *p*>0.05). Interestingly, the systemic NTDs with urinary/adrenal developmental anomalies showed significantly more non-DGV ciliogenic CNVs compared with those without such anomalies (42.3%% *vs* 18.9%, *p*<0.05, one-tailed Fisher exact test). The common urinary/adrenal abnormalities included renal agenesis/hypoplasia, adrenal gland agenesis/hypoplasia and polycystic kidney. This analysis revealed a possible role of cilia genes in some specific systemic NTDs. Table 5 listed detailed phenotypes of 11 systemic NTD cases with non-DGV ciliogenic CNVs and abnormal urinary/adrenal development.

**Table 4:** The Relationship of NTDs categories with non-DGV ciliogenic CNVs.

	With non-DGV ciliogenic CNV (%)	Without non-DGV ciliogenic CNV (%)	*p* value
**Cranial/spinal NTDs**			
** Cranial NTDs**	11 (21.6%)	40 (78.4%)	
** Spinal NTDs**	10 (29.4%)	24 (70.6%)	0.410*
**Isolated/systemic NTDs**			
** Systemic NTDs**	18 (28.6%)	45 (71.4%)	
Abnormal urinary/adrenal gland development	11 (42.3%)	15 (57.7%)	
Normal urinary/adrenal gland development	7 (18.9%)	30 (81.1%)	0.040***
** Isolated NTDs**	3 (13.6%)	19 (82.6%)	0.250**
**Total**	21	64	

* compared with cranial NTDs, two-tailed;

** compared with systemic NTDs, two-tailed;

*** compared with systemic NTDs with abnormal urinary/adrenal gland development, one-tailed.

**Table 5:** The detailed phenotypes in 11 systemic NTDs carrying ciliogenic CNVs and abnormal urinary/adrenal development.

Case ID	The phenotypes	ciliogenic CNV count	cilia gene count	Affected cilia genes and functions	Category of ciliogenicCNVs (kb)	PCR Validation
Case 1	Open spina bifida in lumbar region, bilateral adrenal gland hypoplasia, atrial septal defect	One	One	GLIS3: functions as both a repressor and activator oftranscription and is specifically involved in the development of pancreatic beta cells, the thyroid, eye, liver and kidney. Mutations in this gene have been associated with neonatal diabetes and congenital hypothyroidism (NDH).	Duplication (107 kb)	Figure S1
Case 2	Craniorachischisis, bilateral adrenal gland hypoplasia	Two	Three	CENPN: bound to centromeres throughout interphase and during mitosis. Small interference RNA (siRNA)-mediated depletion of CENPN disrupted recruitment of the core complex, caused the loss of CENPM from centromeres, and increased the number of cells in mitosis.	Deletion (288 kb)	
				C16orf61: Function is not unknown	Deletion (288 kb)	
				PARD6G: Adapter protein involved in asymmetrical cell division and cell polarization processes. May play a role in the formation of epithelial tight junctions. The PARD6-PARD3 complex links GTP-bound Rho small GTPases to atypical protein kinase C proteins	Duplication (58 kb)	Figure S1
Case 3	Spina bifida occulta in lumar- scaral region, hydrocephaly, gastroschisis, congenital talipes equinovarus, bilateral pyelectasis	One	Two	CAMK1D: encodes a member of the Ca2+/calmodulin-dependent protein kinase 1 subfamily of serine/threonine kinases. The encoded protein may be involved in the regulation of granulocyte function through the chemokine signal transduction pathway.	Duplication (531 kb)	
				CDC123: required for S phase entry of the cell cycle (By similarity)	Duplication (531 kb)	
Case 4	Open spina bifida in thoracic and lumbar regions, bilateral adrenal gland hypoplasia	Two	Six	PARP12: catalyzes the post-translational modification of proteins by the addition of multiple ADP-ribose moieties. PARP transfers ADP-ribose from nicotinamide dinucleotide (NAD) to glu/asp residues on the substrate protein, and also polymerizes ADP-ribose to form long/branched chain polymers. PARP inhibitors are being developed for use in a number of pathologies including cancer, diabetes, stroke and cardiovascular disease.	Deletion (47 kb)	
				RAB19: proposed to participate in processes (protein transport, small GTPase mediated signal transduction). Proteins are expected to have molecular functions (GTP binding, nucleotide binding) and to localize in various compartments (cytoplasm, plasma membrane).	Deletion (47 kb)	
				SLC37A3: proposed to participate in processes (carbohydrate transport, transmembrane transport). Proteins are expected to localize in various compartments (endoplasmic reticulum membrane, extracellular space, integral to membrane).	Deletion (47 kb)	
				SLC17A5: encodes a membrane transporter that exports free sialic acids that have been cleaved off of cell surface lipids and proteins from lysosomes. Mutations in this gene cause sialic acid storage diseases, including infantile sialic acid storage disorder and and Salla disease.	Deletion (530 kb)	
				CD109: encodes a member of the alpha2-macroglobulin/complement superfamily. The encoded GPI-linked glycoprotein is found on the cell surface of platelets, activated T-cells, and endothelial cells. The protein binds to and negatively regulates signaling of transforming growth factor beta (TGF-beta).	Deletion (530 kb)	
				C6orf147, C6orf221, C6orf150: function are unknown	Deletion (530 kb)	
Case 5	Open spina bifida in lumbar region, hydrocephaly**,** left kidney agenesis, right polycystic kidney	Two	Two	ACTR3B: Plays a role in the organization of the actin cytoskeleton. May function as ATP-binding component of the Arp2/3 complex which is involved in regulation of actin polymerization and together with an activating. nucleation-promoting factor (NPF) mediates the formation of branched actin networks. May decrease the metastatic potential of tumors	Duplication (899 kb)	
				CTNNA3: recruited E-cadherin and beta-catenin to cell-cell contacts, play role in cell-cell adhesion and tight junction	Duplication (299 kb)	
Case 6	Craniorachischisis, left pyelectasis	One	One	CDC16: required for cell division, cell proliferation and mitotic progression	Duplication (40 kb)	
Case 7	Craniorachischisis, bilateral adrenal gland hypoplasia, atrial septal defect	One	One	ATP5G1: encodes a subunit of mitochondrial ATP synthase. Mitochondrial ATP synthase catalyzes ATP synthesis, utilizing an electrochemical gradient of protons across the inner membrane during oxidative phosphorylation	Deletion (39 kb)	
Case 8	Craniorachischisis, bilateral adrenal gland hypoplasia, cleft lip and palate	One	One	PARD3: involves in asymmetrical cell division and direct polarized cell growth, tight junction via PARD3-PARD6G-aPKC. Seems to play a central role in the formation of epithelial tight junctions, Required for establishment of neuronal polarity and normal axon formation in cultured hippocampal neurons	Deletion (139 kb)	Figure S1
Case 9	Craniorachischisis, left pelvis, ureter and adrenal gland hypoplasia	One	One	STOML3: proposed to participate in a process (signal transduction). Proteins are expected to have molecular function (protein binding) and to localize in various compartments (membrane, integral to membrane, membrane raft, plasma membrane). Putative protein interactors have been described (ADCY3, CAV2ANDCAV1).	Deletion (96 kb)	
Case 10	Craniorachischisis, bilateral adrenal gland malformation	One	Two	ZNF512: proposed to participate in a process (regulation of transcription). Proteins are expected to have molecular functions (DNA binding, GTP binding, metal ion binding, nucleoside-triphosphatase activity and 2 others) and to localize in various compartments (cytoplasm, membrane, nucleus, intracellular). Putative protein interactors have been described (ARMCX3, C19ORF2, CCT4, CCT5, CCT6A, CCT8, DDB1, DHRSXANDZBED1, DHRSXANDZBED1.1, EFTUD2 and 28 others).	Duplication (454 kb)	
				CCDC121: Proteins are expected to localize in nucleus. This gene's function is yet unknown	Duplication (454 kb)	
Case 11	Open spina bifida in thoracic and lumbar regions, hydrocephaly, right kidney and ureter agenesis	One	One	KIAA1586: proposed to participate in a process (regulation of transcription). Proteins are expected to have molecular functions (DNA binding, metal ion binding, zinc ion binding) and to localize in various compartments (nucleus, intracellular). Putative protein interactors have been described (CALCOCO2, FXR2, PRPF40A, RAP1GDS1, RNF40, SNIP1, UBE2I).	Deletion (31 kb)	

**Figure 3:**
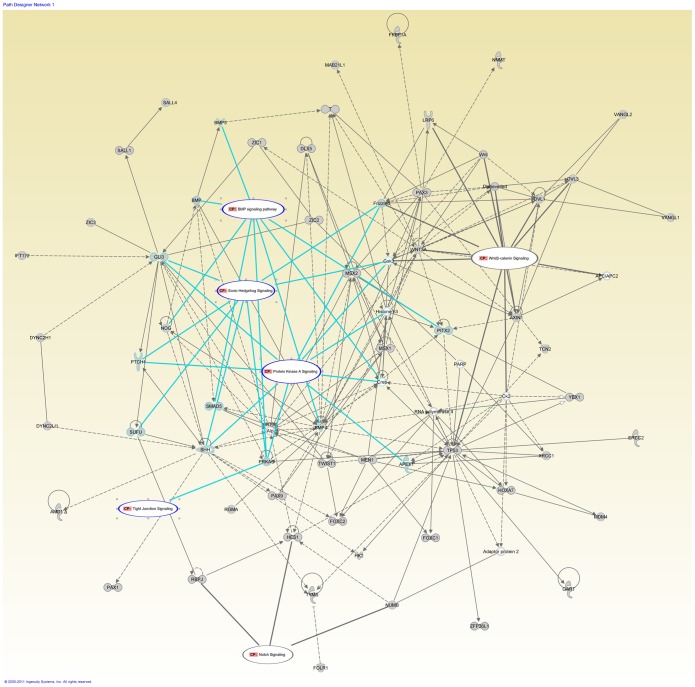
Top bio-functional networks in known NTD candidate genes. Pathways analysis identified “*Organ Development, Embryonic Development, Tissue Development*” in 223 known NTD candidate genes after literatures review. The known signaling pathways for neural tube closure including BMP signaling, SHH and Wnt/β-catenin signaling interact with two novel pathways in NTD-affected cases (CP in the oval regions).

We performed an IPA for 223 known NTD candidate genes. The top network and related diseases/disorders were similar to what was identified with the NTD-specific genes. “*Embryonic Development, Neural System Development and Function, Organ Development*” and “*Amino Acide Metabolism, Molecular Transport, Small Molecular Biochemistry”* were the top networks, and *“Developmental and Neurological Diseases”* were the top related diseases/disorders. “One carbon pool by folate” and “Methionine Metabolism” were the canonical pathways of “*Amino Acid Metabolism*”. For the network *“Embryonic Development, Neural System Development and Function, Organ Development”,* the canonical pathways of known importance to neural tube closure, including sonic hedgehog signaling (SHH), bone morphogenic protein (BMP) signaling, Wnt/β-catenin signaling work were identified in this top network (Fig. 3, labeled as CP). Also we identified tight junction signaling and protein kinase A signaling interact with SHH and BMP signaling (Fig. 3, labeled as CP) by sheared molecules *PRKAC*.

**Figure 4:**
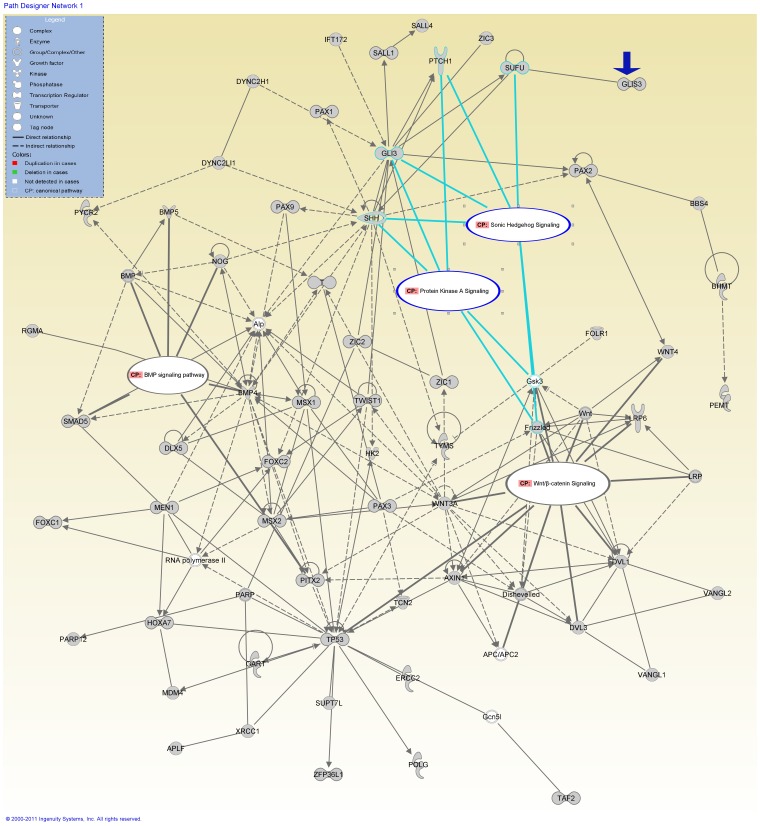
Top bio-functional networks in the integrated list of 361 genes. “*Organ Development, Tissue Development, Embryonic Development*” was identified as top network in the integrated list of 361 genes. The interactions exist between the novel pathway and the known pathway of NTDs.

IPA analysis was performed for 361 integrated genes consisted of known NTD candidate genes and 138 genes from NTD-specific CNVs. We observed that “*Organ Development, Tissue Development, Embryonic Development*” were in the top networks (Fig. 4), and *“Developmental and Neurological Disorders”* was the top related diseases/disorders. 8 genes from NTD-specific CNVs (*APLF, GLIS3, PARP12, POLG, PYCR2, SUPT7L, TAF2, WNT4*) involved in this top network. The SHH, BMP and Wnt/β-catenin signaling and protein kinase A signaling were identified in this network (Fig. 4, labeled as CP).

## Discussion

### Association of Non-DGV Ciliogenic CNVs with NTDs

CNVs are copy number gains or losses of DNA segments ranging from one kilobase to multiple megabases in length that are important underlying factors for human genetic and phenotypic diversity [35]. Genomic CNVs contribute significantly to both rare and common disorders [36], [37], [38], [39], [40]. Previous studies showed that 6.5% of prenatally detected NTDs are associated with chromosome abnormalities using traditional cytogenetic methods [22]. These cytogenetic studies suggested that genomic imbalances could play a significant role in the pathogenesis of NTDs. The rates of chromosomal abnormalities detected by cytogenetic methods differ significantly in different type of NTDs, e.g. 2.3% in anencephaly, 7.1% in encephalocele, and 10.2% in meningomyelocele [22]. It is consistent across several studies that encephalocele have higher chromosomal abnormality rate than anencephaly, and spina bifida have higher chromosomal abnormality rate than craniorachischisis [41], [42], [43]. Among all the detected chromosomal abnormalities, trisomy was the most common chromosomal abnormality associated with NTDs [43]. Those reported chromosomal abnormalities detected by karyotyping are very large (>5 Mb) and helped little to narrow down causal genes for NTDs. Recent microarray-based genomic profiling technologies enabled us to detect smaller CNVs with great sensitivity and specificity. This study is based on the hypothesis that submicroscopic genomic CNVs are genetic risk factors for some NTDs and detection of small CNVs will help us to pinpoint novel NTD candidate genes and related developmental pathways.

To our knowledge, this is the first such study to evaluate the impact of genomic CNVs on the occurrence of NTD in Chinese patients. As a result, we detected many small CNVs in NTD samples. Not surprisingly most of these CNVs were reported in the DGV database and are presumably benign polymorphisms. A subset of non-DGV CNVs in NTD samples is novel and not shared with CNVs observed in unrelated clinical patient cohorts, suggesting that they are potentially NTD-specific, important for identifying causal genes for NTDs. Due to the lack of parental samples, we were not able to evaluate their *de novo* status. Instead, we compared the collective frequencies of non-DGV, particularly genic CNVs in cases and controls. Enrichment of ciliogenic CNVs was identified as a risk factor for human NTD. The significant enrichment of genic CNVs in NTD compared to controls is very similar to what was discovered in schizophrenic patients [29]. Xu et al. compared the distribution of genic CNVs in 200 schizophrenia individuals and 159 matched controls. Significant higher enrichment of rare genic CNVs was seen in familial schizophrenia than controls (relative enrichment = 2.7, *p* = 10^−3^). But unlike what was found in schizophrenia patients, we failed to identify any recurrent CNVs in NTDs that pose high penetrant effect on the phenotype.

### Enriched Ciliogenic CNVs in NTDs and Ciliogenesis Pathways in Neural Tube Closure

Computerized functional analyses for genes encompassed in common/rare CNVs have been previously performed to reveal their potential roles in disease-susceptibility [29], [36], [44]. Common CNVs often encompass the “environmental sensor” genes. These genes are not necessarily critical for early embryonic development, but rather enable environmental adaptation. Such adaptive genes include olfactory receptors, immune and inflammatory response genes, cell signaling molecules and cell adhesion molecules [45]. In contrast, genes disrupted by rare CNVs in neurodevelopmental patients were significantly involved in psychological disorders and learning behaviors [36], [44]. Our study revealed that different functional pathways are involved with genic CNVs detected in case and control samples. In control samples, the top diseases were related to immunological and infectious diseases, which are similar to what were found in common CNVs. By contrast, genes related to neurological and genetic disorders were overrepresented in NTD groups, reflecting the neurodevelopmental nature of the neural tube closure process.

The signaling pathways known to be essential for neural tube closure include PCP signaling (non-canonical Wnt pathway), SHH, BMP, Wnt and Notch signaling and inositol phosphorylation metabolism [7]. Recently primary ciliogenesis processes have been proved to be important for primary neurulation via interaction with the SHH pathway [46], [47]. NTDs are frequent phenotypes in ciliary dysfunction disorders [48]. The overlap of developmental pathways and phenotypes implies a potential role for cilia-related genes in human NTDs. In this study, we identified the enrichment of ciliogenic CNVs in NTD cases. Moreover, two canonical pathways (tight junction signaling and protein kinase A signaling) identified from NTD-specific CNVs are primary cilio-related pathways [48], [49] and show interaction with known NTD pathways. Our results support the notion that some genes encompassed by NTD-specific CNVs are functionally related to ciliogenesis pathways and are potentially responsible for the NTDs.

Among cilia genes identified in NTD-specific CNVs, *GLIS3* (MIM 610192) is a member of the GLI-similar zinc finger protein family and a nuclear transcription regulator. Mutations and a large deletion in the 5-UTR region of the *GLIS3* gene have been reported in children with neonatal diabetes mellitus and congenial multiple malformation including polycystic kidney [50]. In addition, the association of *GLIS3* CNVs with diabetes was reported [51]. Interestingly, diabetes and gestational diabetes are recognized risk factors for NTDs [52]. Case 1 in this study was an embryo with open spina bifida and bilateral adrenal gland hypoplasia, atrial septal defect. It carried a single rare duplication covering the *GLIS3* 5-UTR and exon 1 (see Table 5 for phenotype, see Figure S1 for microarray picture and validation). Functional network analysis demonstrated that *GLIS3* participates in the network involved in NTD pathogenesis (see Figure 2 and 4 for its role in top networks, blue arrowheads). *PARD3-PARD6* (MIM 606745) has been proved to be essential for epithelial polarity during embryogenesis and neurulation [53]. This polarity complex also localizes to cilia and regulates ciliogenesis, tight junction during kidney morphogenesis [54]. Zebrafish model revealed that *PARD3* is critical in orchestrating the midline crossing during neurulation [55]. *PARD3* depletion in zebrafish results in intracellular cilia and hydrocephalus [56]. human *PARD3/PARD6* knock-out in MDCK cell resulted in severe morphogenetic defect on three-dimensional culture condition, forming multiple lumen which is similar to polycystic kidney phenotype in human [57]. The case carrying *PARD3/PARD6G* CNVs (case 8 and case 2 in Table 5, see Figure S1 for microarray picture and validation) presented with adrenal gland hypoplasia in addition to craniorachischisis. In case 8, *PARD3* was the only gene in the sole non-DGV CNV detected. With the available maternal samples, we excluded the maternal inheritance of *PARD3* deletion in case 8. *CTNNA3* (MIM 607667) recruits E-cadherin and beta-catenin to cell-cell contacts, and plays role in cell-cell adhesion and tight junction. Its homolog *ctnnbip1* was reported as candidate gene of mouse NTDs [9]. The mice with mutant *ctnnbip1* presented with exencephaly (equivalent to anencephaly in human). Case 5 carried *CTNNA3* and *ACTR3B* duplication, and exhibited open spina bifida, kidney agenesis and polycystic kidney phenotype. *ACTR3B* duplication is possible genetic factor to explain why the NTD phenotype in case 5 was different from *ctnnbip1-*mutated mouse. The maternal inheritance of *CTNNA3* and *ACTR3B* duplication was excluded by its mother’s blood.

### Complex Genetic Heterogeneity of NTDs in Shanxi Province

We enrolled NTDs cases from Shanxi Province, which has the highest NTD prevalence in the world. This is a region that was not supplemented with folic acid at the time of sample collection. Previous studies supported that low folate levels, and polymorphism of folate-metabolic genes were risk factors for NTDs in this region [58], [59], [60]. Genes regulating early embryonic development were also identified as risk factors of NTDs in this region [61], [62], [63]. The significant involvement of copy number variants in NTD revealed by this study further suggests the complex genetic heterogeneity underlying NTD pathogenesis in the Shanxi region. We noticed two unique features pertaining to our NTD cohort. 1) None of the cases harbored CNVs larger than 3 Mb, the majority of CNVs were between 100–500 kb in size and no trisomy was detected in this not previously screened NTD cohort. Previous studies identified large chromosomal abnormalities including trisomy in a significant percentage of NTD cases among patients from Western countries [21], [22], [23], [41], [42]; this phenomenon may indicate underlying genetic differences between our cohort collected in Shanxi region and cases collected for previous studies. 2) The NTD cases collected for this study consisted of many cases with additional malformations outside the nervous system. For example, 63 cases were categorized as systemic NTDs. Our finding of the enrichment of cilia genes in NTD related CNVs is consistent with the pleiotropic roles of cilia genes in the development of different systems. Among systemic NTD subtype, the borderline significance between systemic NTDs accompanying abnormal urinary/adrenal gland development and ciliogenic CNVs may reflect their unique genetic basis. Adrenal hypoplasia/agenesis have been reported as associated morphologic anomalies in fetus with NTDs, and 29–57% NTD cases were found to have low adrenal weights [64], [65], [66]. Although the role of cilia genes on adrenal gland development is unknown, knock out of cilia genes (*PARD3/PARD6*) in mammary stem/progenitor cells can result in severe morphogenetic defects, forming multiple lumens in the mammary gland [67]. Future studies with larger sample size will help to verify the association between systemic NTDs, urinal/adrenal malformation and cilia genes.

In conclusion, we detected the enrichment of genic CNVs, particular ciliogenic CNVs in NTD cases relative to controls, suggesting the association of ciliogenic CNVs with NTD. Pathway analysis further revealed that two ciliogenesis pathways including tight junction and protein kinase A signaling are involved with the pathogenesis of human NTDs.

## Supporting Information

Figure S1 **Examples of six rare genic CNVs and their validations by PCR.** A–F show three heterozygous deletions and three heterozygous duplications. A–C represent the 139 kb deletion in the PARD3 region (chr10∶34875595-35015198), the 30 kb deletion in the NRG3 region (chr10∶84631526-84661716), and the 42 kb deletion in the SLIT2 region (chr4∶20030784-20086137). D–F show three heterozygous duplications with different sizes. They are a 636 kb duplication in the PRKX region (chrx:3528099-4164677), a 107 kb duplication in the GLIS3 region (chr9∶4142060-4249876), and a 58 kb duplication in the PARD6G region (chr18∶76009131-76067279). G–I show the validations of the CNVs by long-range PCR and real-time quantitative PCR. G and H present bilateral breakpoints of A and B validated by long-range PCR following sequencing. Sequences were blasted (USCS hg18) online to get an exact joint site. I shows the real-time PCR validation for C–F. A normal male without these CNVs was used as control sample. CNVs were calculated using log 10 of CNV ratio (the height of case/mean height of control sample). The minus index represents deletions and the plus index represents duplications. For each CNV, three pairs of primers pairing to the head, middle and tail regions were used to amplify three segments.
(TIF)

Table S1 **Primers for validating six non-DGV genic CNVs by long-range/real-time PCR.**
(XLS)

Table S2 **Different categories of non-DGV CNVs and non-DGV genic CNVs in the two groups.**
(XLS)

Table S3 **Gene list compassed in non-DGV CNVs from NTD samples.**
(XLS)

Table S4 **Gene list compassed in non-DGV CNVs from matched controls.**
(XLS)

Table S5 **Known candidate gene list of human/mouse NTDs.**
(XLS)

Table S6 **Primers and enzymes of MKS1/MKS3 PCR-sequencing.**
(XLS)
