# Supplementary figures and images for: Detection of Copy Number Variants Reveals Association of Cilia Genes with Neural Tube Defects
Source: PLoS One. 2013 Jan 17;8(1):e54492. doi: 10.1371/journal.pone.0054492 (PMC3547935; doi:10.1371/journal.pone.0054492)

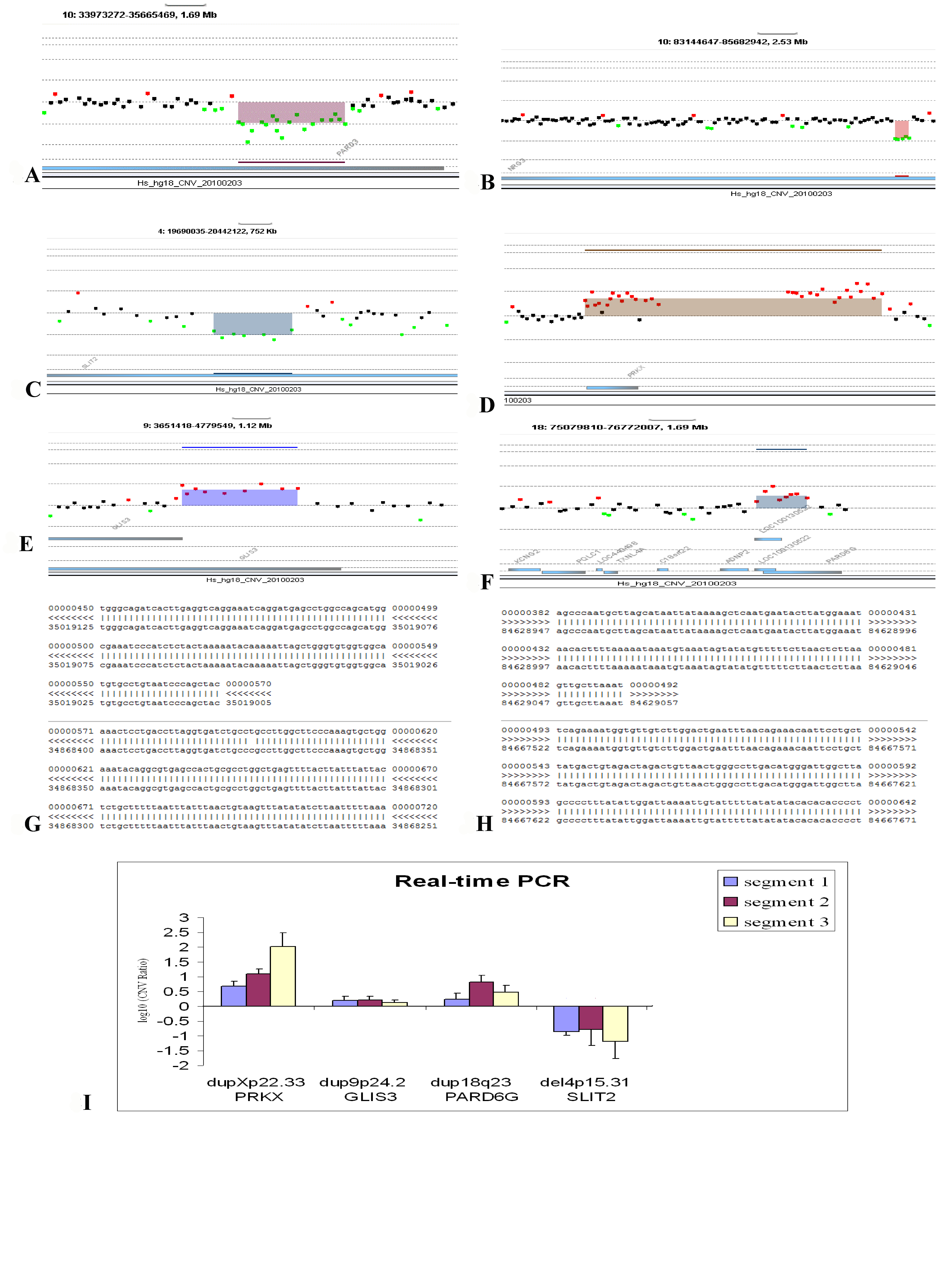

Supplement: Figure S1 — Examples of six rare genic CNVs and their validations by PCR. A–F show three heterozygous deletions and three heterozygous duplications. A–C represent the 139 kb deletion in the PARD3 region (chr10∶34875595-35015198), the 30 kb deletion in the NRG3 region (chr10∶84631526-84661716), and the 42 kb deletion in the SLIT2 region (chr4∶20030784-20086137). D–F show three heterozygous duplications with different sizes. They are a 636 kb duplication in the PRKX region (chrx:3528099-4164677), a 107 kb duplication in the GLIS3 region (chr9∶4142060-4249876), and a 58 kb duplication in the PARD6G region (chr18∶76009131-76067279). G–I show the validations of the CNVs by long-range PCR and real-time quantitative PCR. G and H present bilateral breakpoints of A and B validated by long-range PCR following sequencing. Sequences were blasted (USCS hg18) online to get an exact joint site. I shows the real-time PCR validation for C–F. A normal male without these CNVs was used as control sample. CNVs were calculated using log 10 of CNV ratio (the height of case/mean height of control sample). The minus index represents deletions and the plus index represents duplications. For each CNV, three pairs of primers pairing to the head, middle and tail regions were used to amplify three segments. (TIF) [file pone.0054492.s001.tif]
